# Anaerobic hydrolysis of complex substrates in full-scale aerobic granular sludge: enzymatic activity determined in different sludge fractions

**DOI:** 10.1007/s00253-021-11443-3

**Published:** 2021-07-24

**Authors:** Sara Toja Ortega, Mario Pronk, Merle K. de Kreuk

**Affiliations:** 1 Section Sanitary Engineering, Department of Water Management, Delft University of Technology, Stevinweg 1, Delft, 2628 CN The Netherlands; 2 Department of Biotechnology, Delft University of Technology, van der Maasweg 9, Delft, 2629 HZ The Netherlands; 3 Royal HaskoningDHV, Laan 1914 35, Amersfoort, 3800 AL The Netherlands

**Keywords:** Aerobic granular sludge, Full-scale, hydrolysis, Domestic wastewater, Polymeric substrates

## Abstract

**Abstract:**

Complex substrates, like proteins, carbohydrates, and lipids, are major components of domestic wastewater, and yet their degradation in biofilm-based wastewater treatment technologies, such as aerobic granular sludge (AGS), is not well understood. Hydrolysis is considered the rate-limiting step in the bioconversion of complex substrates, and as such, it will impact the utilization of a large wastewater COD (chemical oxygen demand) fraction by the biofilms or granules. To study the hydrolysis of complex substrates within these types of biomass, this paper investigates the anaerobic activity of major hydrolytic enzymes in the different sludge fractions of a full-scale AGS reactor. Chromogenic substrates were used under fully mixed anaerobic conditions to determine lipase, protease, α-glucosidase, and β-glucosidase activities in large granules (>1 mm in diameter), small granules (0.2–1 mm), flocculent sludge (0.045–0.2 mm), and bulk liquid. Furthermore, composition and hydrolytic activity of influent wastewater samples were determined. Our results showed an overcapacity of the sludge to hydrolyze wastewater soluble and colloidal polymeric substrates. The highest specific hydrolytic activity was associated with the flocculent sludge fraction (1.5–7.5 times that of large and smaller granules), in agreement with its large available surface area. However, the biomass in the full-scale reactor consisted of 84% large granules, making the large granules account for 55–68% of the total hydrolytic activity potential in the reactor. These observations shine a new light on the contribution of large granules to the conversion of polymeric COD and suggest that large granules can hydrolyze a significant amount of this influent fraction. The anaerobic removal of polymeric soluble and colloidal substrates could clarify the stable granule formation that is observed in full-scale installations, even when those are fed with complex wastewaters.

**Key points:**

• *Large and small granules contain >70% of the hydrolysis potential in an AGS reactor.*

• *Flocculent sludge has high hydrolytic activity but constitutes <10% VS in AGS.*

• *AGS has an overcapacity to hydrolyze complex substrates in domestic wastewater.*

**Graphical abstract:**

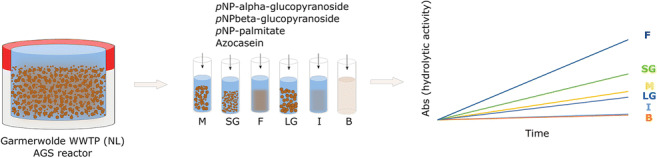

**Supplementary Information:**

The online version contains supplementary material available at 10.1007/s00253-021-11443-3.

## Introduction

In recent years, aerobic granular sludge (AGS) technology has emerged as an alternative to the conventional activated sludge (AS) technology for the treatment of domestic wastewater (Bengtsson et al. 2018; Pronk et al. 2015). AGS is composed of granular microbial aggregates and can remove chemical oxygen demand (COD), phosphorus (P), and nitrogen (N) from wastewater (Bassin et al. 2012; Coma et al. 2012; de Kreuk et al. 2005; Layer et al. 2019). AGS requires less space and energy than AS, due to the granular morphology that improves the settling properties of the sludge, which leads to a smaller footprint and a simple reactor design. The technology is applied by Royal HaskoningDHV at full-scale under the trade name Nereda^®^, based on a sequencing batch reactor (SBR) cycle consisting of simultaneous anaerobic feeding and effluent removal, aeration, and settling (Giesen et al. 2013; Pronk et al. 2017). Full-scale AGS, fed with domestic wastewater, operates stable and can remove COD and nutrients efficiently (Giesen et al. 2014; Ni et al. 2009; Pronk et al. 2017). Still, some aspects of full-scale operation of AGS need further study to optimize this novel process and understand the observations made in pilot and full-scale applications. One main knowledge gap is the removal mechanism of influent particulate COD.

The composition of the influent is a main difference between full-scale practice and most lab-scale experiments. Substantial knowledge about AGS has been gained studying lab-scale reactors fed with simple influents, rich in volatile fatty acids (VFA) (Bassin et al. 2012; de Kreuk et al. 2005; Mosquera-Corral et al. 2005; Weissbrodt et al. 2014; Zeng et al. 2003). The conditions in these studies promoted the growth of slow-growing, storage polymer-forming microorganisms, such as polyphosphate accumulating organisms (PAO). Slow-growing microorganisms contribute to granule formation and stability (de Kreuk and van Loosdrecht 2004; Picioreanu et al. 1998). The selected PAO population additionally removes phosphorus and nitrate from the wastewater, therefore contributing to enhanced biological phosphorus removal (EBPR). Since acetate and propionate are the main substrates of PAOs, their loading rate is an important factor for EBPR treatment plant design and operation (Lopez-Vazquez et al. 2020).

VFA content of domestic wastewater is highly variable, ranging as much as from 1 to 50% of the total influent COD. The VFA concentration can be influenced by several parameters such as type of wastewater, sewer type, temperature, and residence time in the sewer (Hvitved-Jacobsen et al. 1995; Narkis et al. 1980; Rudelle et al. 2011; Yun et al. 2013). Most domestic wastewaters contain 10% or less VFA-COD, while the remaining COD is composed of complex substrates, such as proteins, carbohydrates, and lipids (Levine et al. 1991; Raunkjær et al. 1994; Rudelle et al. 2011; Volcke et al. 2020). They are present in the wastewater as particulates (e.g., cellulose fibers, microbial cells) or as colloid and soluble polymers. In AGS systems, a large fraction of the particulate COD ends up in the flocculent sludge fraction in the reactor (Guo et al. 2020; Layer et al. 2019). This fraction has a relatively short solids retention time (SRT) (Ali et al. 2019) reducing the mineralization of the particulate COD (Guo et al. 2020). On the other hand, polymeric substrates could be used for EBPR and granule growth, which would be beneficial for AGS reactors treating wastewaters with a low or highly variable VFA content. During the anaerobic feeding phase, easily degradable soluble COD can be stored cell-internally as poly-hydroxyalkanoates (PHA), while polymeric substrates can potentially be hydrolyzed and fermented to VFA and stored sequentially. Hydrolysis has often been described as the rate-limiting step in biological degradation of organic matter (Balmat 1957; Ubukata 1997). Hence, in AGS reactors, the hydrolysis of polymeric substrates during the feeding phase will determine to a large extent the amount of substrate that is available for PHA storage and thus for the bio-P removal.

Moreover, incomplete uptake of polymeric substrates during the anaerobic phase, and their presence in the aerobic phase, is often associated with irregular sludge morphology, decreased settleability, or smaller granule size (de Kreuk et al. 2010; Derlon et al. 2016; Layer et al. 2019; Wagner et al. 2015). Therefore, it is relevant for good AGS performance to have sufficient hydrolytic capacity for a complete transformation of the polymeric substrates in the influent during the anaerobic phase. Although efforts have been made to characterize hydrolysis in biofilm systems at lab scale (Kommedal et al. 2006; Mosquera-Corral et al. 2003), and AS systems at lab- and full-scale (Frølund et al. 1995; Goel et al. 1998; Morgenroth et al. 2002), hydrolysis in full-scale AGS reactors has not been extensively studied. Furthermore, the hydrolytic potential of aerobic granules, its accompanying flocculent biomass, and the activity in the bulk liquid are still unknown.

To evaluate the hydrolytic potential of polymeric substrates under the anaerobic feeding conditions, we measured the activities of specific hydrolytic enzymes of the sludge derived from a full-scale Nereda^®^ installation. The sludge fractions were incubated with chromogenic lipase, protease, and glucosidase substrates under anaerobic fully mixed conditions and were sampled periodically to monitor hydrolysis rates. The hydrolytic activity of the mixed sludge, large (>1 mm) and small (0.2–1 mm) granules, flocculent sludge, influent wastewater, and bulk liquid of the AGS reactor was determined and evaluated considering the composition of the incoming wastewater. Large granules were crushed and their activity was measured to study the effect of mass transfer limitation on hydrolysis. By studying anaerobic hydrolytic activity, this study aimed to elucidate the involvement of different types of biomass in the anaerobic conversion of polymeric COD, in AGS systems treating domestic wastewater.

## Materials and methods

### Biomass sampling and separation

Biomass and influent were sampled at wastewater treatment plant (WWTP) Garmerwolde (The Netherlands) in the period from April 30 to May 9, 2019. Another sampling round was performed in February 2020 to measure granule size distribution and conduct additional enzyme assays. The stable operation of the plant and the low variability of the sludge volume index (SVI_5_) between the two sampling periods led to the assumption that the granule bed composition was comparable between both periods. The characteristics of WWTP Garmerwolde are described by Pronk et al. (2015). Two Nereda^®^ reactors, designed by Royal HaskoningDHV (Amersfoort, The Netherlands), treat 35,000 m^3^ day^−1^ on average, half of the total flow to the wastewater treatment plant. The influent was sampled after screening and grit removal, and two types of influent samples were collected for different purposes: (1) Three representative 24-h flow-proportional samples were collected and used for physical-chemical characterization of the influent; (2) For each hydrolytic assay, influent grab samples were collected, to ensure that the influent sample used in the enzyme assays was as fresh as possible. The grab samples were used in the hydrolytic assays, and not characterized in terms of chemical composition. The sludge samples were taken from the AGS reactor at least 40 min after the start of the mixed aeration phase to ensure a homogeneous sample. Samples were preserved at 4°C, and the activity tests were performed within 8 h after the sample was taken.

For the hydrolytic activity tests, 7 sample types were prepared: mixed sludge, large granules, crushed large granules, small granules, flocs, bulk liquid, and influent. The sludge was washed over a stack of sieves by applying tap water with moderate pressure. The following fractions were collected: large granules (>1000 μm in diameter), small granules (200–1000 μm in diameter), and flocs (45–200 μm in diameter). A lower limit was used for flocs to separate them from the bulk liquid. Crushed granules were obtained by dispersing 15 g of large granules using a Potter-Elvehjem-type tissue grinder. The bulk liquid fraction was obtained by settling the sludge for 2 h and collecting the supernatant. Mixed sludge was diluted to approximately 4 g/L using 20 mM Tris-HCl buffer. All the other fractions were also buffered with 20 mM Tris-HCl and diluted to a final approximate concentration of 4 g mixed liquor suspended solids (MLSS)/L. The activity of the influent, on α- and β-glucosidase assays, was determined using a raw influent sample. For the protease and lipase assays, the influent particles were concentrated by settling: 2 L of influent was settled and the supernatant was discarded to keep a final volume of 250 mL. This concentration step was needed to increase the signal in the protease assay, which was less sensitive than the other assays. The influent used in lipase assays was also settled, because the same samples as for protease assays were used for lipase assays, since they were performed on the same days. Complementary experiments were performed in February 2020 to compare the activity of the settled and non-settled influents. The pH of all the samples was set to 7.5 for the assay.

### Hydrolytic activity tests

The substrates used for hydrolytic activity tests were *p*-nitrophenyl (*p*NP) conjugates (*p*NP-palmitate, *p*NP-α-glucopyranoside and *p*NP-β-glucopyranoside), and azocasein. These substrates target lipase, α-glucosidase, β-glucosidase, and protease activities, respectively. All used chemicals were purchased from Sigma-Aldrich (Darmstadt, Germany). Excess concentrations of substrate were used in the assay to ensure zero-order kinetics and thus to measure maximum hydrolytic activity. *p*NP-α-glucopyranoside, *p*NP-β-glucopyranoside, and azocasein solutions were prepared by dissolving the powder substrates in Tris-HCl. These substrates were fully dissolved. *p*NP-palmitate, however, was insoluble in Tris-HCl buffer, so an emulsion was prepared in an isopropanol-Tris-HCl mixture (Supplementary information: Fig. S1). The tests were performed in 40-mL vials with air-tight stoppers equipped with a sampling port. On each assay, all the sample types derived from one sampling were run in parallel. The vials were flushed with N_2_ for 1 min to create anaerobic conditions. The temperature of the assays was between 18 and 20°C. Biomass was incubated with the chromogenic substrates on a Fisherbrand Seastar orbital shaker from Thermo Fisher Scientific (Waltham, MA USA) at 120 rpm, and samples were taken regularly throughout the length of the experiment. The samples were sieved through a 100-μm mesh to remove biomass. Immediately after, 1 mL of sample was mixed with 1 mL of 30% (w/v) trichloroacetic acid (TCA), to stop the enzymatic reaction. Samples were stored at −20°C until analyzed. The frozen samples were thawed at room temperature, centrifuged, and filtered through 0.45 μm. One milliliter of sample was mixed with 1 mL of 2 M NaOH. Finally, absorbance was measured in a Genesys 10S UV-Vis spectrophotometer from Thermo Fisher Scientific (Waltham, MA, USA) at the corresponding wavelength (Table 1).

**Table 1:** Chromogenic substrates and assay conditions were used for the different enzyme assays

Assay	Substrate	Concentration	Assay length	Absorbance measured
Lipase	*p*NP-palmitate	20 mM	1 h 15 min	410 nm
α-Glucosidase	*p*NP-α-glucopyranoside	1 mM	1 h 15 min	400 nm
β-Glucosidase	*p*NP-β-glucopyranoside	1 mM	1 h 15 min	400 nm
Protease	Azocasein	0.2 % (w/v)	2 h 30 min	440 nm

### Calculation of hydrolytic activity

Absorbance was plotted over time and the data was analyzed through linear regression. Samples with an *R*^2^ value lower than 0.7 were discarded (Lundstedt et al. 1998), considering that the activity was below the detection limit of the method. Samples with less than 4 data points (due to sample loss or problems in sample analysis) were also discarded. Triplicate samples were averaged and the standard deviation was calculated aggregating the standard deviation between triplicates and the standard deviation of the fits. The slope of the regression was used to calculate the hydrolytic activity of the samples.

To couple increase in absorbance to moles of substrate hydrolyzed, substrate characteristics were taken into account. *p*NP-conjugated substrates release one *p*NP mol per mol of substrate hydrolyzed, and therefore, the activity is expressed as micromole *p*NP per hour. A calibration curve with known *p*NP concentrations was used to translate absorbance values to micromole *p*NP. Protease activity is generally reported in terms of tyrosine equivalents: 1 U protease = 1 μmol Tyr/min (under certain T and pH). To express protease activity as micromole tyrosine equivalents per hour (μmol Tyr eq h^−1^), a correlation was made between absorbance at 440 nm and Tyr equivalents, for azocasein (Supplementary information: Fig. S2). The Tyr content of azocasein is 6.9 Tyr per mol of protein, and therefore, 1 mol Tyr equivalent would translate to 0.14 moles of protein hydrolyzed.

The specific hydrolytic activity of the different types of sludge was calculated by dividing the measured activity by the amount of biomass used in the assay. Total activity at reactor level contributed by the different sludge fractions was calculated by multiplying the specific activity by the amount of each type of sludge in the reactor. For influent and bulk fractions, the specific activity was expressed per volume unit. Their total enzyme activity at reactor level was calculated by multiplying the specific activity by the volume of influent fed per cycle, and the volume of bulk in the reactor. Bulk volume was calculated as follows:

$$ {V}_{\mathrm{b}}={V}_{\mathrm{r}}\times \left(1-\mathrm{TS}\times {\mathrm{SVI}}_{30}\times \Big(1-\varepsilon \right)\Big), $$

where *V*_b_ is the volume of bulk, *V*_r_ is the reactor volume, TS is the concentration of sludge in the reactor, and *ε* the porosity of the packed sludge bed, which is assumed to be 0.52 based on van Dijk et al. (2020).

The total amount of substrate that could be degraded during a 1 h anaerobic feeding was calculated simplifying the plug-flow into 10 CSTRs (continuous stirred-tank reactors) over the total fed influent volume. The following assumptions were made: (1) The influent fills up the reactor according to the feeding flow, and the hydrolytic activity of each CSTR segment is only considered once the influent reaches it. (2) The settled sludge bed occupies the volume corresponding to its SVI_5_ of 40 mL/g. This is around 3830 m^3^ of sludge, while the average feed batch is 3870 m^3^ based on the average flow and a feeding of one h. Thus, at the end of the feeding the whole sludge bed is filled with influent. (3) The sludge bed is stratified due to differences on settling velocity of the different granule sizes (van Dijk et al. 2020). Considering the composition of the sludge bed (see the “Biomass composition” section), the first 9 segments only contain large granules, and the last segment is a mixture of large granules, small granules, and flocculent sludge. The total hydrolytic activity calculated this way was translated from micromole *p*NP per hour and micromole tyrosine equivalents per hour to mg COD substrate hydrolyzed. To do so, we considered the molecular weight of the substrates, the ratio of dye released to moles of substrate hydrolyzed mentioned above, and the COD per mg substrate for each biomolecule type based on Sophonsiri and Morgenroth (2004).

### Analysis of the biomass

Total solids (TS) and volatile solids (VS) of the biomass were measured following Standard Methods (APHA 2005). Granule size distribution measurements were performed sieving 1.5 L of sludge through the following mesh sizes: 1000 μm, 200 μm, and 45 μm. The total solids and volatile solids of the resulting sludge fractions were determined as described in the Standard Methods. The weight percentage of each faction with respect to the total was calculated.

A VHX-700F digital microscope from Keyence (Mechelen, Belgium) was used to take micrographs of the biomass used for the experiments. The dimensions of the large granules were determined via image analysis using the built-in software of the microscope. The average granule diameter values given by the software were used to calculate the sphere-equivalent volume of the granules. Particle size distribution of the flocculent sludge and crushed granules was measured using a Bluewave light-scattering particle size analyzer from Microtrac (Montgomeryville, USA).

### Analysis of the influent wastewater

Twenty-four-hour flow-averaged influent wastewater samples were preserved at 4°C for up to 24 h. Biological oxygen demand (BOD_5_) was measured using photochemical test kits, with product number LCK 555, from Hach (Düsseldorf, Germany). Total suspended solids (TSS) and volatile suspended solids (VSS) of the influent were measured as described in the Standard Methods (APHA 2005). Part of the influent was filtered applying positive pressure through a 0.45 μm pore size, using a cross-flow filter, to keep a soluble influent fraction. The raw and filtered influent were stored at −20°C until further use.

The stored influent samples were thawed at room temperature in closed vials. Soluble and total COD were analyzed using photochemical test kits, product number LCK514, from Hach (Düsseldorf, Germany). Soluble and total protein concentration were measured using the modified Lowry method (Frølund et al. 1995) which distinguishes proteins and humic compounds. Soluble and total carbohydrate concentration were measured using the anthrone-sulfuric acid method (DuBois et al. 1956). Lipid content was measured using the gravimetric determination method by Merieux Nutri-Sciences (Resana, Italy). The measured concentrations of proteins, lipids, and carbohydrates were converted to COD based on Sophonsiri and Morgenroth (2004).

Long-term measurements of total COD, BOD_5_, TSS, total phosphorus (TP), phosphate (PO_4_^3−^-P), total nitrogen (TN), and ammonium (NH_4_^+^-N) in the wastewater were performed by a certified lab and provided by the water authority Noorderzijlvest (Groningen, The Netherlands).

## Results

### Influent wastewater composition

Several influent parameters were analyzed on three sampling days. The results of the analyses are summarized in Table 2. In addition, long-term measurements of the wastewater composition are shown, as provided by the water authority Noorderzijlvest. These averaged values were obtained from the regular plant monitoring in the period between 1 April 2019 and 6 February 2020.

**Table 2:** Composition of the influent wastewater to the Nereda^®^ reactor. Long-term measurement results were provided by the water authority and resulted from regular plant monitoring (April 2019 to February 2020)

	Measurements during sampling campaign					Long-term measurements	
	01-05-2019	07-05-2019	08-05-2019	26-02-2020 ^a^	Average	Average	Lowest–highest
tCOD (g m^−3^)	567	703	587	301	619	544	260–800
sCOD (g m^−3^)	143	133	127	-	134	-	-
BOD_5_ (g m^−3^)	129	238	283	-	217	231	100–370
TSS (g m^−3^)	393	445	384	-	407	248	140–380
VSS (g m^−3^)	220	308	172	-	233	-	-
TP (g m^−3^)	-	-	-	-	-	6.6	2.5–12
PO_4_^3−^-P (g m^−3^)	-	-	-	-	-	4	1.6–5.9
TN (g m^−3^)	-	-	-	-	-	49	21–73
NH_4_^+^-N (g m^−3^)	-	-	-	-	-	41	16–59
Soluble proteins (g COD m^−3^)	13	18	21	-	17	-	-
Total proteins (g COD m^−3^)	66	86	52	-	68	-	-
Soluble carbohydrates (g COD m^−3^)	13	9	8	-	10	-	-
Total carbohydrates (g COD m^−3^)	110	210	109	-	143	-	-
Soluble humics (g COD m^−3^)	99	65	67	-	77	-	-
Total humics (g COD m^−3^)	157	101	148	-	135	-	-
Lipids (g COD m^−3^)	22	-	-	17	20	-	-
Volatile fatty acids (g COD m^−3^)	58	9	40	-	29	-	-

^a^The sample on 26-02-2020 was taken during wet weather flow (WWF) and is not used to calculate the average COD concentration

The rounded average flow to the two Nereda^®^ reactors during the experimental period was 31,000 m^3^ day^−1^. With a biological reactor volume of 9,500 m^3^ per reactor, the volumetric loading rate of each reactor was 1.6 m^3^ (m^3^ day)^−1^. The average sludge loading approximated 0.07 kg COD (kg TS day)^−1^. The reactor was operated at an average volumetric exchange ratio (VER) of 40%.

### Biomass composition

**Fig. 1:**
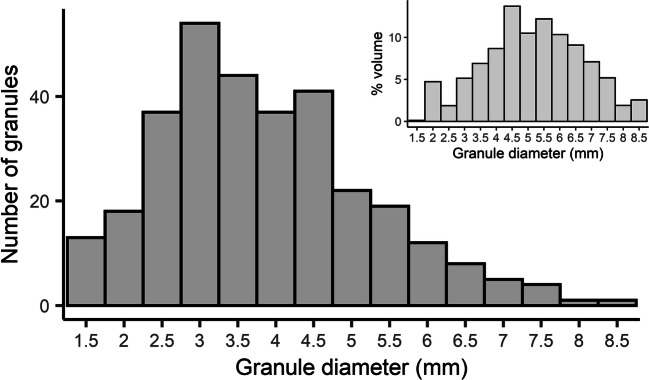
Granule size distribution of the large granule fraction measured by image analysis. *N* = 317 granules. On the top right corner, calculated volume distribution of large granules (sphere-equivalent volume)

The biomass concentration of the reactor during the sampling period was between 12 and 15 g TS/L. The VS/TS ratio of the sludge was 80 ± 1%. The SVI_5_ during this period was 30–40 mL/g. The proportion of large granules (> 1 mm) in the reactor was remarkably high in this plant; they accounted for 84% of the VS. Small granules were 7% of the VS and flocculent sludge 9%. The distribution of diameters of large granules (>1 mm) is shown in Fig. 1. The average diameter of the large granule fraction is 3.6 mm. However, it should be noted that when accounting for the volume occupied by the granules of the different sizes, the distribution shifts towards larger diameters.

The separation of the biomass using sieves did not render perfect separation of the biomass fractions (Supplementary information: Fig. S3). The small granule fraction (0.2–1 mm) was highly heterogeneous and consisted of a fiber/cellulose matrix that entrapped biomass and inorganic particles of different sizes. This made it impossible to determine the granule size of this sludge fraction using the image analysis software.

Flocculent sludge (0.045–0.2 mm) and crushed granules had a comparable particle size distribution (Supplementary information: Fig. S4), showing a thorough homogenization of the large granules.

### Hydrolytic enzyme activity distribution in the sludge

All mixed sludge samples showed hydrolysis activity on all substrates tested. The specific activities for the enzymes measured are summarized in Table 3. It should be taken into account that the dye released per substrate hydrolyzed is not equal for all assays (see Materials and methods), and therefore, the hydrolysis rates of the different substrates cannot be compared directly.

**Table 3:** Specific hydrolytic activities of the mixed sludge sample

	Hydrolytic activity: μmol *p*NP (g VS h)^−1^ ; μmol Tyr eq (g VS h)^−1^	stdev	n
α-Glucosidase	13	2	9
β-Glucosidase	17	5	8
Lipase	34	26	5
Protease	10	6	9

**Fig. 2:**
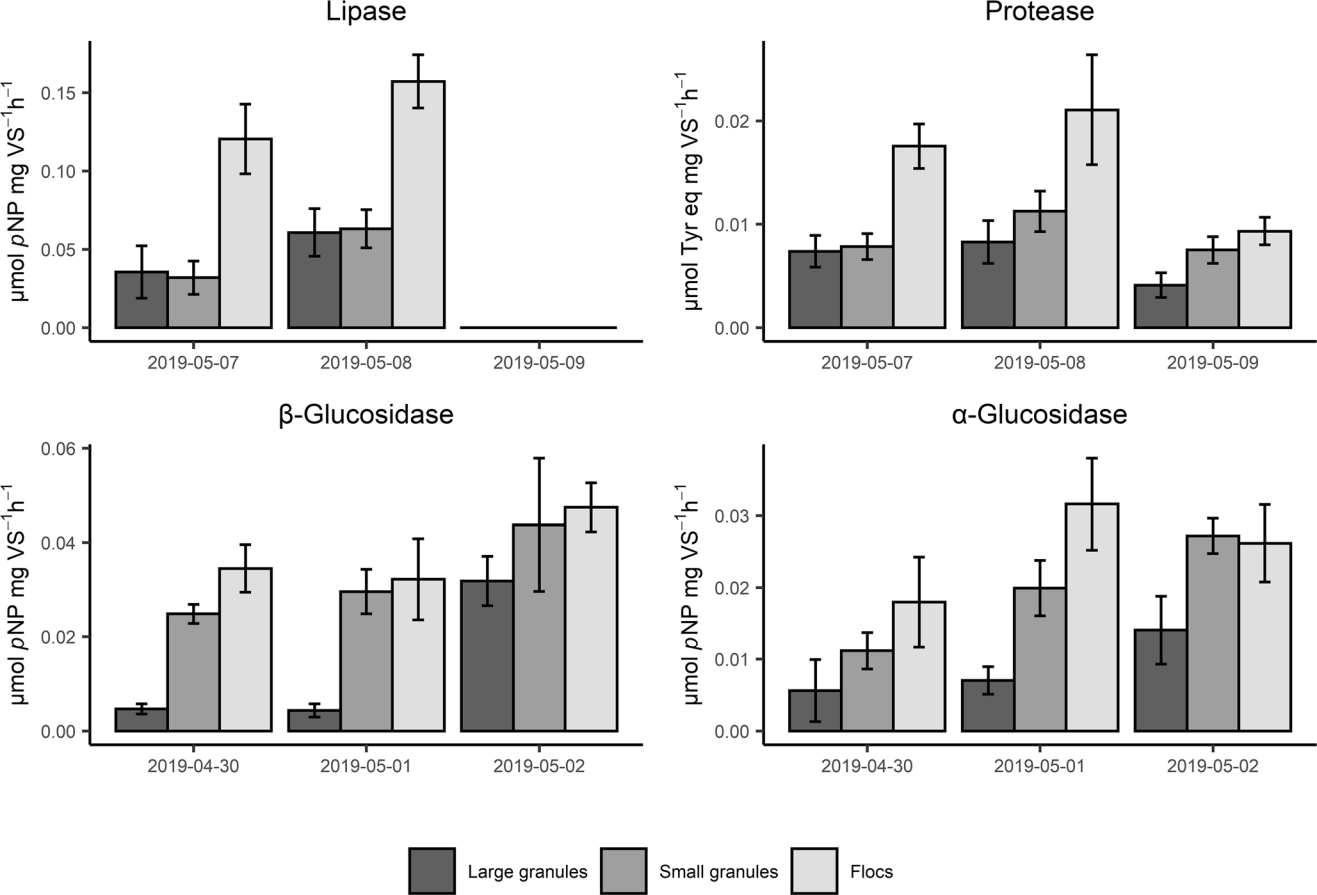
Specific hydrolytic activities measured in the different sampling days. Note that lipase samples from the 9th of May had to be discarded due to poor fit of the linear regression. Each bar represents triplicate samples and error bars represent the standard deviation of the triplicates

The distribution of the activity of hydrolytic enzymes in the different sludge fractions is shown in Fig. 2. The maximum hydrolyzed amounts during an anaerobic feed of 1 h, estimated with the measured hydrolytic activities, would be 570 mg COD/L of protein, 650 mg COD/L of lipid, 80 mg COD/L of α-glycosides, and 160 mg COD/L of β-glycosides (240 mg COD/L of carbohydrates). The biomass-specific activity of all the hydrolytic enzymes tested was highest in the flocculent sludge fraction, while granules exhibited lower hydrolytic activity per gram VS. The difference between these fractions differed per type of enzyme and sampling day. Flocculent sludge activity was 1.5 to 7.5 times higher than large granule activity. Depending on the enzyme group and sampling day, the activity of the small granule fraction was comparable to the larger granules, between that of flocculent sludge and large granules, or comparable to the flocculent sludge.

**Fig. 3:**
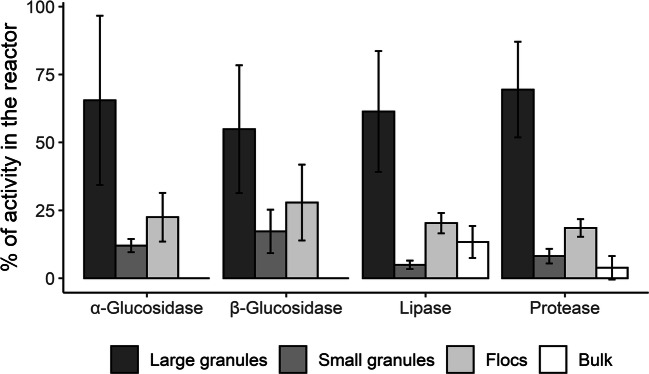
Percentage of hydrolytic activity contributed by large granules, small granules, flocs, and bulk liquid with respect to the total reactor activity. The values represented in the graph are averaged over 3 days of sampling (2 days in the case of lipase). Error bars represent standard deviation

The hydrolytic activity of each sludge fraction at reactor level was calculated by multiplying the hydrolytic activity per gram VS by the total VS mass of that fraction within the reactor. The bulk liquid activity was determined as activity per milliliter and multiplied by the total volume of the bulk liquid in the reactor. Figure 3 shows the percentage of enzyme activity contributed by each of the biomass fractions, averaging the data from all the sampling days. The data for each of the days can be found in Supplementary information: Fig. S5. Considering the total mass of large granules in the reactor, the total enzyme activity of the large granule fraction was higher than that of flocs and small granules. Reactor level hydrolytic activity of the granule fraction was 2–3.8 times higher than that of the flocculent fraction for all the substrates. It is noteworthy that flocculent sludge still had relatively high hydrolytic potential on a reactor scale (18–28% of the total), even though flocculent sludge occupied only a small fraction of the VS in the reactor.

α- and β-glucosidase activities were not detected in the bulk liquid. Bulk protease activity was detected but contributed only 4% of the total protease activity in the reactor. Besides, there were large differences in the bulk protease activity measured per day (Supplementary information: Fig. S5). Lipase activity was rather high in the bulk liquid, accounting for 13% of the activity in the reactor. Upon the observation of high bulk lipase activities, an additional experiment was conducted centrifuging the bulk liquid at 12,000 rpm for 15 min, to ensure that all the cells and other particles were removed from the sample. The centrifuged bulk still contained high lipase activity, although it was 20% lower than the activity of the non-centrifuged bulk. This result indicated that indeed, the bulk activity measured in the assays was mainly due to enzymes in solution and not due to cell-bound enzymes.

### Hydrolytic activity of the influent

**Fig. 4:**
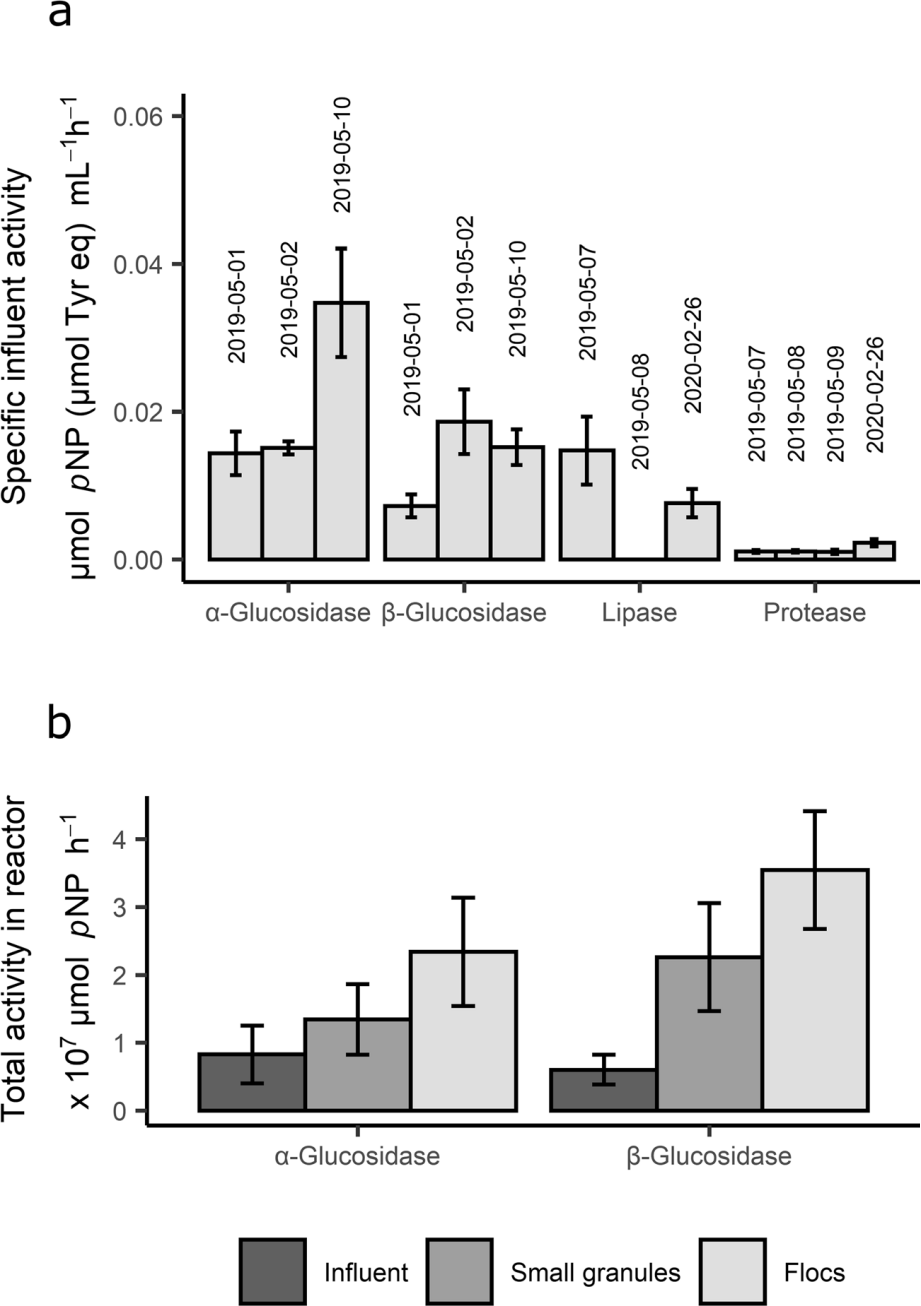
Hydrolytic activity of the influent wastewater. **a** Specific influent hydrolytic activities (activity/mL) measured in different sampling days. Lipase and protease activities were measured in a settled influent sample; the activity shown in the graph is the calculated activity per mL of the original influent, taking the settling step into account. Each bar represents triplicate samples. Error bars represent the standard deviation of the triplicates. **b** Comparison of α- and β-glucosidase activities contained in one feed batch (3,870 m^3^) with the total activity present in the small granules and flocculent sludge in the reactor. Each bar represents the average activity during the different sampling days, and error bars represent standard deviation. Values for large granules and bulk liquid are not included in this figure, but their activity relative to small granules and flocs can be found in Fig. 3

All the enzymes measured showed activity in the influent too (Fig. 4a). Considering an average influent feed of 3,870 m^3^/cycle, the influent hydrolytic activity that is fed to the reactor per cycle was calculated. In comparison to the sludge activities measured, the activity carried by the influent was rather high in the case of α- and β-glucosidase (Fig. 4b). The α-glucosidase activity brought by the influent was, on a reactor level, nearly as high as that of small granules.

Lipase activity was underestimated in the assays where the influent particles were concentrated by settling. An assay comparing the non-settled influent to the settled influent showed that the activity contributed by the settled influent fraction was only 43% of the total activity of the wastewater. Therefore, more than half of the lipase activity in the wastewater was in the supernatant. Thus, concentration of the influent by settling should be avoided when determining its hydrolytic potential, since the soluble activity can be quite significant. The protease activity of the total influent could not be measured due to lower sensitivity of the protease assays, and thus, it is unclear if influent protease assays also neglected the activity in the supernatant.

### Enzyme activity in dispersed sludge

**Fig. 5:**
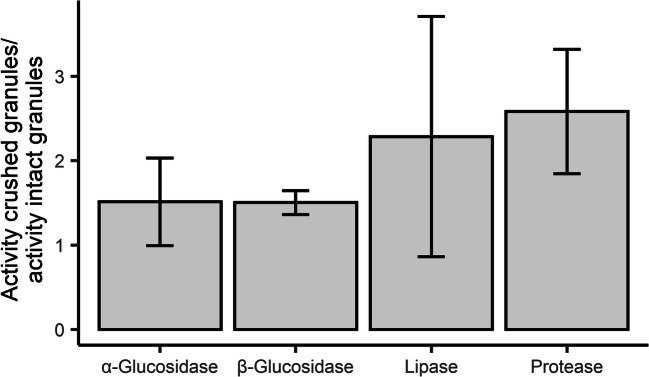
Activity of crushed granules over activity of intact large granules. Each bar represents three experiments performed in triplicate (except in lipase where the data is from two experiments, also in performed in triplicate). Error bars represent standard deviation

Comparison of the enzymatic activity of intact and crushed granules was used to account for diffusion limitation of the substrates towards the inside of the granules (Fig. 5). The activity of crushed granules was higher than that of full granules. This difference was larger in the case of lipase and protease; intact granules only exhibited 40 to 44% of the protease and lipase activity measured in the crushed granules.

A higher variability was observed in lipase activity than in the other enzyme activities, which might be due to differences in the level of solubilization of the *p*NP-palmitate solution used in each assay. A poorly dissolved palmitate would render higher observed differences between intact and crushed granules. The absorbance values of the *t*_0_ samples highly differed per day, showing high heterogeneity during substrate preparation.

## Discussion

### Sample heterogeneity and daily variations

The present study disclosed high variability in the determination of hydrolytic activity of AGS fractions and influent wastewater. This could be for several reasons. One of them is the substrate preparation procedure, which in the case of *p*NP-palmitate is laborious. The other substrates were directly prepared in Tris-HCl and there were no problems with solubilization. With *p*NP-palmitate, however, the slightest difference in preparation conditions (e.g., stirring speed, time, and speed of addition of the reagents to the solution) affected the characteristics of the final substrate solution, resulting in inconsistent degrees of solubilization. Different concentrations of soluble substrate on different assays might be a reason behind the high variability of the measured lipase activity between days. Apart from the implications on measurement reproducibility, this also indicates that the form in which the substrate is found in the wastewater (i.e., particulate or soluble) will greatly affect its hydrolysis rate.

Another source of variability was the heterogeneity of the sludge. The standard deviations of the triplicate samples were significantly high, in some cases reaching 30–40% of the average value. The differences between triplicates were highest in the large granule fraction. Taking a representative sample of aerobic granules is challenging, due to difficulties in sampling fast-settling granules, and also due to the size heterogeneity of the granule fraction. As shown in Fig. 1, the large granule fraction consisted of a large range of diameters. Considering the relatively low volumes of sludge used in the assay (40 mL per vial), the different granule diameters might not have been equally represented in all replicates. Therefore, the use of high granule quantities is advisable to diminish this variability between samples, as well as setting an upper size limit for large granules, in order to keep surface to volume ratios of the granules in the sample similar. The quantity of crushed granules prepared was 500 mL, making sure a representative granule size distribution was used for these experiments.

Lastly, daily fluctuations in the influent composition and concentration added most likely to the variability of the measurements. Hydrolytic activities tend to vary due to differences in influent composition, environmental conditions, and reactor operation (Nybroe et al. 1992). Large variations between days were observed on the measured hydrolytic activity, as shown in Figs. 1 and 3. Similarly to our observations, high long-term variations in enzyme activities of AS have been previously reported (Frølund et al. 1995). The relative contributions of the different types of sludge to the total activity fluctuated less than the individual activities of the fractions. This enabled us to draw general conclusions about the enzyme activity distribution in the AGS reactor studied. Other studies have reported different sludge bed compositions, with smaller granule size and higher concentrations of flocculent sludge fractions (Cetin et al. 2018; Derlon et al. 2016; Layer et al. 2019; van Dijk et al. 2018). Based on our results, it is clear that the proportions of the different granules and floc sizes affect the overall hydrolytic activity of the reactor, and therefore, these fractions need to be reported to be able to compare different reactor performance datasets.

### Biomass-bound activity

This study showed that different sludge fractions from a full-scale AGS reactor have the potential to hydrolyze influent polymers under anaerobic conditions. Most of the hydrolyzing potential was found to be biomass-bound for all the substrates, but lipase activity was also rather high in the reactor bulk liquid (13% of the total activity). Since the bulk liquid was separated from its suspended solids by settling, low density or buoyant lipid aggregates might have been retained in the bulk liquid sample, including the attached lipases.

For proteins and carbohydrates, hydrolytic activity in the reactor is mainly bound to the biomass. Larsen and Harremoës (1994) reported high bulk liquid hydrolysis of carbohydrates, but most AS and biofilm studies assigned the main contribution to hydrolysis and particle removal to the biomass (Boczar et al. 1992; Boltz and La Motta 2007; Confer and Logan 1998; Frølund et al. 1995; Karahan et al. 2006; Mosquera-Corral et al. 2003). Frølund et al. (1995) described AS flocs as a matrix of immobilized enzymes. This allows the retention of enzymes inside the reactor, and due to the lifetime of enzymes, they can be active in a different phase of the cycle than they were synthesized (Boczar et al. 1992; Goel et al. 1998). The retention of enzymes would support the hypothesis that aerobic and anaerobic hydrolyses occur at similar rates. This would justify using one hydrolytic constant while modeling AGS, regardless of the redox conditions in the reactor, as proposed in the activated sludge model no. 3 (ASM3) (Gujer et al. 1999). Yet, degradation of polymers by protozoa or other predators should be considered separately, since their activity was not researched in this work and might differ during anaerobic and aerobic conditions.

Having enzyme activity predominantly in the (granular) biomass fraction also implies that contact between the polymeric substrates and biomass is necessary for their hydrolysis. Recent studies employing magnetic resonance imaging (MRI) and mass balancing explored the substrate-sludge interaction during a plug-flow passage of influent through a settled bed of AGS (Layer et al. 2020; Ranzinger et al. 2020). Ranzinger et al. (2020) concluded that colloidal substrates are evenly distributed throughout the granular sludge bed during plug-flow feeding, but that the interaction between particulates larger than 1 μm and AGS was limited. Even though it is difficult to extrapolate small-scale flow experiments to full-scale reactors that are 45 m in diameter, we could argue that colloidal and polymeric (soluble) substrates, like the substrates used in our assays, are accessible to the granule bound enzymes in the granules during anaerobic plug-flow feeding. In contrast, the fate of larger particulate substrates in full-scale systems remains unclear. The high hydrolytic potential in granules found in the present study encourages further research on how different types and sizes of organic particles exactly interact with granular biomass during feeding, and to what extent they are available to the hydrolytic enzymes. This insight could increase the effectiveness and duration of the anaerobic period, increasing the overall reactor efficiency.

### Surface-dependent hydrolysis

Large granules (>1 mm in diameter) were able to hydrolyze all substrates tested. Their specific activity, however, was lower than that of small granules (0.2–1 mm) and flocculent sludge (0.045–0.2 mm). A lower specific activity of large granules was expected due to their low surface area to volume ratio. The diffusion of polymeric substrates in biofilms is highly reduced or even negligible (Carlson and Silverstein 1998), and therefore, their hydrolysis will be influenced by the biofilm surface area. As reported by previous studies, polymer degradation is restricted to the first few micrometers of biofilm surface (de Kreuk et al. 2010; Kommedal et al. 2006). Since azocasein is a polymer with a molecular weight of approximately 23 kDa, the lower specific protease activity of large granules compared to other sludge types can be explained by transport limitation. *p*NP-palmitate is a smaller substrate (378 Da). Nonetheless, the *p*NP-palmitate substrate was an emulsion due to the poor solubility of palmitate in water. (Supplementary information 1). Moreover, palmitate has been reported to adsorb to the surface of anaerobic granules, hindering its diffusion (Palatsi et al. 2012). Therefore, a similar dependency of the surface area to volume ratio for its hydrolysis could be expected. The α- and β-glucosidase substrates used in these assays had a molecular weight of 301 Da. Substrates of this size (smaller than sucrose) can diffuse into biofilms rather easily (Stewart 1998). Nevertheless, flocculent sludge had higher α- and β-glucosidase activity than large granules and crushed granules had 1.5 times the activity of intact granules. This suggests that even for small, soluble substrates, the observed hydrolysis rate is strongly affected by the transport of the substrates into the granules. The surface area available for hydrolysis will thus be crucial for the hydrolysis of most substrates, but most importantly for substrates of larger size which cannot diffuse into granules.

The results of lipase and protease assays with crushed granules also evidence surface-limited hydrolysis in large granules. By dispersing the granules and removing the resistance to substrate diffusion, the hydrolysis rate increased considerably. The hydrolytic enzymes made available by crushing might be located in deeper layers of the granule and only have access to the assay substrates upon crushing. Therefore, they are likely not involved in the degradation of influent substrates in situ. These enzymes could be degrading complex substrates in the granule matrix, such as polymers embedded in the granules or products from cell decay (Adav et al. 2009). Alternatively, the enzymes released when crushing granules could be mainly located close to the surface and hydrolyze influent substrates, but at reduced rates, due to limiting substrate concentrations below the surface induced by mass transfer resistance. Knowing more specifically where hydrolyzing enzymes are located in granules would help to understand the processes happening inside the granule, and approximate which layer of the granules, and thus fraction of the biomass, contributes to the conversion of complex influent substrates.

### Hydrolytic activity of the influent

The influent contained α- and β-glucosidase activities in the same order of magnitude as the sludge fractions; for example, α-glucosidase activity of the influent was about 1/3 of that of flocs. Besides, soluble carbohydrate concentrations in the influent were low: readily biodegradable carbohydrates were probably hydrolyzed in the sewer prior to the arrival to the plant. The remaining particulate carbohydrates might keep hydrolyzing during feeding to the reactor; during a 1-h feeding period, a maximum of 10% of the total carbohydrates could be degraded by the enzymes in the influent. A previous study suggested that the hydrolysis of influent suspended solids would be mainly performed by microorganisms in the influent (Benneouala et al. 2017). Besides, a recent study on the microbial composition of the wastewater treatment plant of Garmerwolde identified several hydrolyzing microorganisms in the influent (Ali et al. 2019). Nevertheless, in contrast to AS, the influent microorganisms have a short retention time in the reactor. Large granules share few taxa with the influent; they are composed of a highly specialized, and stable, microbial community with a very high SRT, while the microbial composition of the flocculent sludge fraction is fluctuating and more affected by the changes in influent composition (Ali et al. 2019). This indicates that the hydrolyzing bacteria coming with the influent are likely to end up in flocs when the sludge bed is mixed during the aeration phase; hence, the hydrolytic activity contained in influent particles would be partly discharged with the selection spill and partly stay at the top of the granule bed due to stratification during sludge settling (van Dijk et al. 2020). It is thus unlikely that this layer with flocs would contribute to the hydrolysis of the particles under the anaerobic feeding conditions, during which the influent is fed from the bottom of the reactor, especially considering the applied VER of 40%.

### Full-scale hydrolysis and implications for practice

This study demonstrated that aerobic granules have the potential to significantly contribute to overall reactor hydrolysis. The hydrolytic potential of the large granules determined in this study would be enough to anaerobically degrade much higher concentrations of soluble protein and carbohydrates than those present in the influent (45 and 25 times higher, respectively). The granular sludge also had an overcapacity to hydrolyze the total lipid, protein, and carbohydrates concentrations that were measured in the influent during the anaerobic phase. However, it should be noted that the rates in this study are maximum rates: they were measured at excess substrate conditions, and the complex substrates present in domestic wastewater likely have an overall lower biodegradability than the four model substrates used here. Therefore, the hydrolysis rates measured in this study are not directly applicable to granular sludge models. These hydrolysis rates reflect the enzyme concentration in the different biomass fractions, but not the in situ hydrolysis rate. The hydrolysis constant in granular sludge models should be derived from measurements of the hydrolysis rate of a specific influent by a specific biomass fraction.

In full-scale AGS, during the anaerobic feeding phase influent is fed through the settled sludge bed from the bottom of the reactors in a plug-flow regime (Pronk et al. 2015; van Dijk et al. 2020). The settling velocity of granules is heavily influenced by their size (Liu et al. 2008; Winkler et al. 2012). Consequently, the settled sludge bed is stratified with larger granules at the bottom and smaller ones on top (van Dijk et al. 2020). Due to the lack of mixing during feeding, it is most likely that the products of hydrolysis will only be available close to where hydrolysis takes place. These conditions highlight the importance of having hydrolytic activity associated with the large granules. PAOs from large granules would then have preferential access not only to the already present VFA in the influent, but also to the VFA that are formed in the anaerobic phase. This would mean that higher P removal efficiencies could be achieved than based solely on influent VFA. Knowing that the sludge has the ability to hydrolyze polymers, the length of the anaerobic phase can be adjusted to enhance EBPR performance in AGS reactors with low influent VFA. Our results encourage further in situ research linking hydrolysis rates with anaerobic uptake of substrates in domestic wastewater. Still, the hydrolytic activity in aerobic granules measured in this study suggests that they would have access to more COD during anaerobic feeding than only influent VFA, supporting granule formation, and EBPR.

Anaerobic hydrolysis and uptake of the hydrolysis products are beneficial for the granule morphology too (de Kreuk et al. 2010). If polymeric substrates are present during aeration, hydrolysis will continue and substrate will slowly be released during this phase, creating local substrate gradients at the surface of the granules and favoring fast-growing heterotrophs. This can result in irregular outgrowth of biofilm surfaces and deteriorate the settleability of the sludge (de Kreuk et al. 2010; Mosquera-Corral et al. 2003). Considering the hydrolytic activities measured in this study, most of the soluble and colloidal polymeric substrates will likely be degraded by granules during the anaerobic phase, and will not be present during aeration. This rests on the assumption that soluble and colloidal substrates have sufficient interaction with granules (Ranzinger et al. 2020). The hydrolysis of larger particulate substrates during plug-flow feeding might be limited by their contact with the sludge, as discussed before. Their aerobic hydrolysis will only provide residual readily biodegradable COD (rbCOD) concentrations during aeration. A slow supply of rbCOD during the aerobic phase is mostly linked to flocculent sludge growth, with minimal impact on granule structure given appropriate selective sludge removal is applied (Haaksman et al. 2020). Previous works also observed that reactors fed with non-diffusible substrates developed a fraction of flocculent sludge (Derlon et al. 2016; Layer et al. 2019; Wagner et al. 2015), and Layer et al. (2019) hypothesized that the co-existence of the two morphologies was beneficial for AGS stability. The particles that are not degraded in the aerobic phase but are incorporated in the floc structure can be used during the (optional) anoxic phase as substrates for denitrification. Tougher particles (e.g., cellulose fibers) will not be fully degraded within the SRT of flocs (<7 days) (Ali et al. 2019) and will be removed with the excess sludge during the sludge selection spill (Guo et al. 2020; Pronk et al. 2015). A high content of influent particles in the excess sludge could be related to its high biogas potential due to low mineralization of the spill sludge discharge (Guo et al. 2020). Hydrolysis of easily biodegradable polymers by large granules during the anaerobic phase, and selective removal of the excess sludge containing the more hardly biodegradable substrates, would explain the fairly regular granule growth observed in Garmerwolde (Supplementary information: Fig. S3), and other AGS reactors fed with municipal wastewaters (Cetin et al. 2018; Derlon et al. 2016; van Dijk et al. 2020).

## Supplementary Information

ESM 1 (PDF 1126 kb)

## Data Availability

The datasets generated and/or analyzed during the current study are available from the corresponding author on reasonable request.
