# Supplementary material for: Anaerobic hydrolysis of complex substrates in full-scale aerobic granular sludge: enzymatic activity determined in different sludge fractions
Source: Appl Microbiol Biotechnol. 2021 Jul 24;105(14-15):6073–86. doi: 10.1007/s00253-021-11443-3 (PMC8390406; doi:10.1007/s00253-021-11443-3)
Supplement: Supplementary file 1 — (PDF 1126 kb) [file 253_2021_11443_MOESM1_ESM.pdf]

SUPPLEMENTARY INFORMATION

Applied Microbiology and Biotechnology

Anaerobic hydrolysis of complex substrates in full-scale aerobic granular sludge: enzymatic activity determined in different sludge fractions

Sara Toja Ortega [a], Mario Pronk [b,c], Merle K. de Kreuk [a]

[a] Section Sanitary Engineering, Department of Water Management, Delft University of Technology, Stevinweg 1, 2628CN Delft, The Netherlands.

[b] Department of Biotechnology, Delft University of Technology, Van der Maasweg 9, Delft, 2629, HZ, the Netherlands.

[c] Royal HaskoningDHV, Laan 1914 35, Amersfoort, 3800 AL, The Netherlands.

Corresponding author: Sara Toja Ortega. Email: S.TojaOrtega@tudelft.nl; Telephone number: +34 660699411

S1. Preparation of *p*NP- palmitate solution

The *p*NP- palmitate solution was prepared in isopropanol due to its poor solubility in water, based on Gupta et al. (2002) and Winkler and Stuckmann (1979). The protocol was adapted to prepare a higher concentration *p*NP-palmitate solution, increasing the isopropanol and Triton X-100 concentrations. To prepare 100 mL of a substrate solution of 30 mM *p*NP-palmitate, the following procedure was followed:

1. 0.74 g of sodium deoxycholate (Sigma-Aldrich, Darmstadt, Germany. Product number D6750) were dissolved in 35.8 mL of isopropanol, stirring vigorously using a magnetic stirrer. It is essential to provide sufficient turbulence during this step. The solution should be kept stirring until a dense, white emulsion is reached.
2. 1.01 g *p*NP- palmitate were added to the solution. The solution was kept vigorously stirring until no *p*NP- palmitate flakes are observed.
3. 0.3 g gum Arabic were added to the solution and stirred until dissolved.
4. When a homogeneous suspension was achieved, the solution was transferred to a magnetic stirrer with a heating plate and heated to 35 °C.
5. 53.3 mL Tris-HCl (20 mM, pH 7.5) was slowly added to the suspension, while stirring.
6. 10.7 mL Triton X-100 were added to the final solution.

The resulting *p*NP-palmitate solution was a cloudy solution, as shown in Fig. S1.

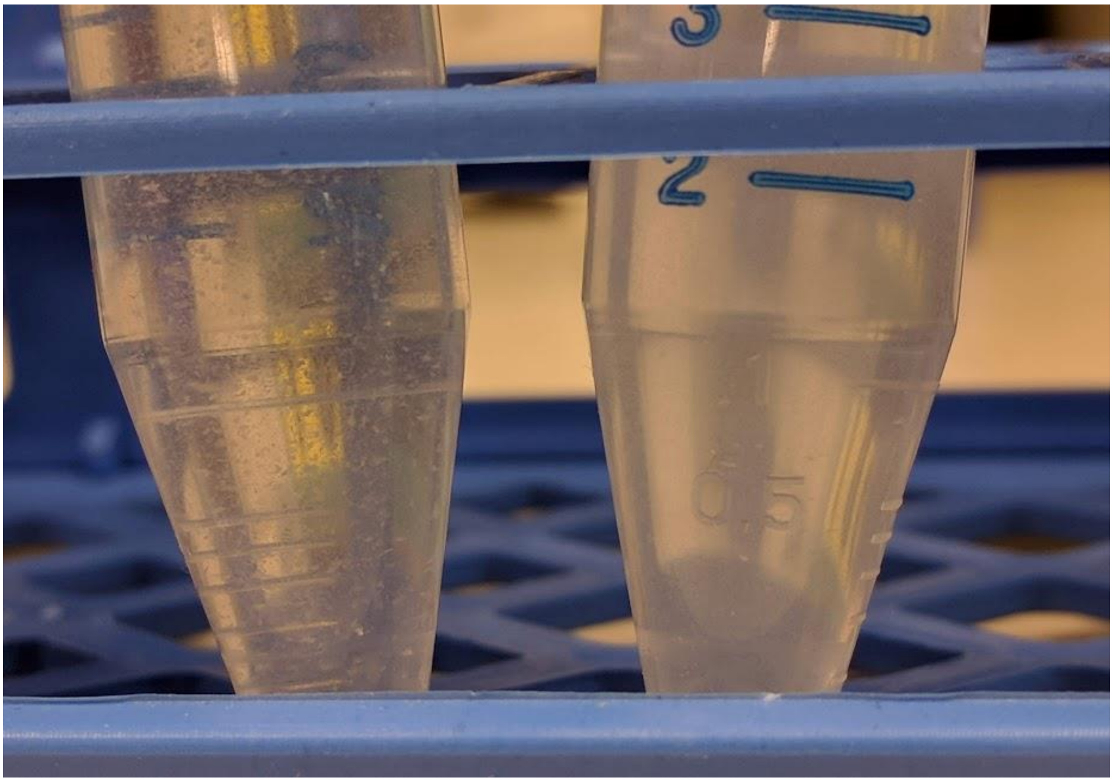

Fig. S1. *p*NP-palmitate solutions, prepared without temperature control (left) and at 35 °C (right). Notice the difference between the cloudy emulsion in the right, and the clear solution with palmitate precipitates in the left.

S2. Protease assays: translation from Absorbance 440 to Tyrosine equivalents

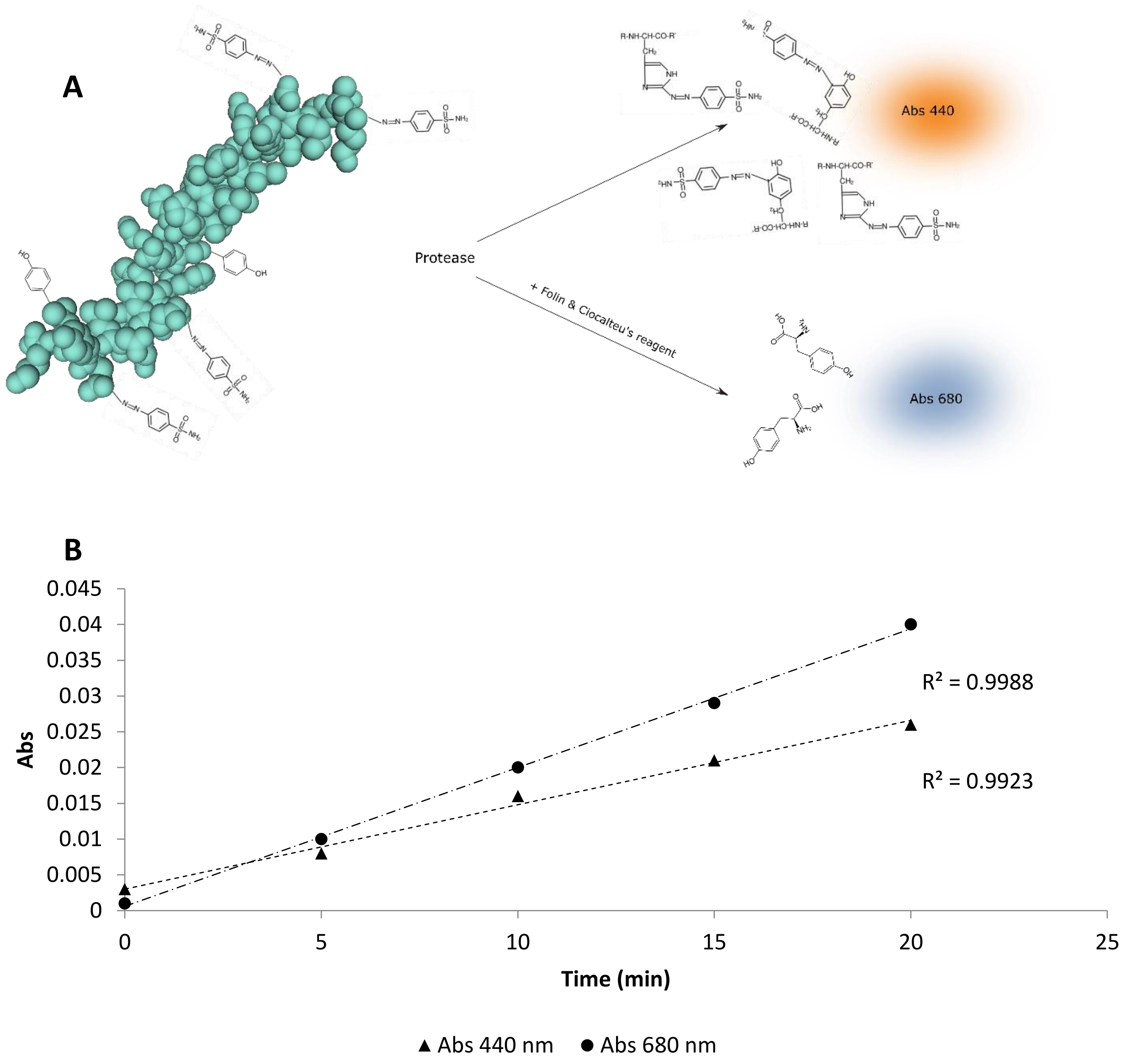

Fig. S2. Procedure to translate Abs 440 to Tyrosine equivalents. A) Azocasein was hydrolysed using a commercial protease (Sigma-Aldrich, Darmstadt, Germany. Product number P4630). The release of tyrosine and azo dye during hydrolysis was measured in parallel. Duplicate samples were taken at several points through time. In one of the samples, absorbance at 440 nm was measured, to detect the azo dye released. The other sample was treated with Folin & Ciocalteu's reagent, which reacts with tyrosine residues making them detectable at Abs 680. Abs 680 was then translated to Tyr equivalents using a calibration curve with L-Tyrosine (Sigma-Aldrich, Darmstadt, Germany. Product number T3754) B) Plot of Abs 440 and Abs 680 through time during the enzymatic reaction. The slopes of Abs 440 vs time and Abs 680 vs time were used to translate from Abs 440 to Abs 680, and later to Tyr equivalents.

S3. Micrographs of the sludge and influent fractions used in the enzyme assays

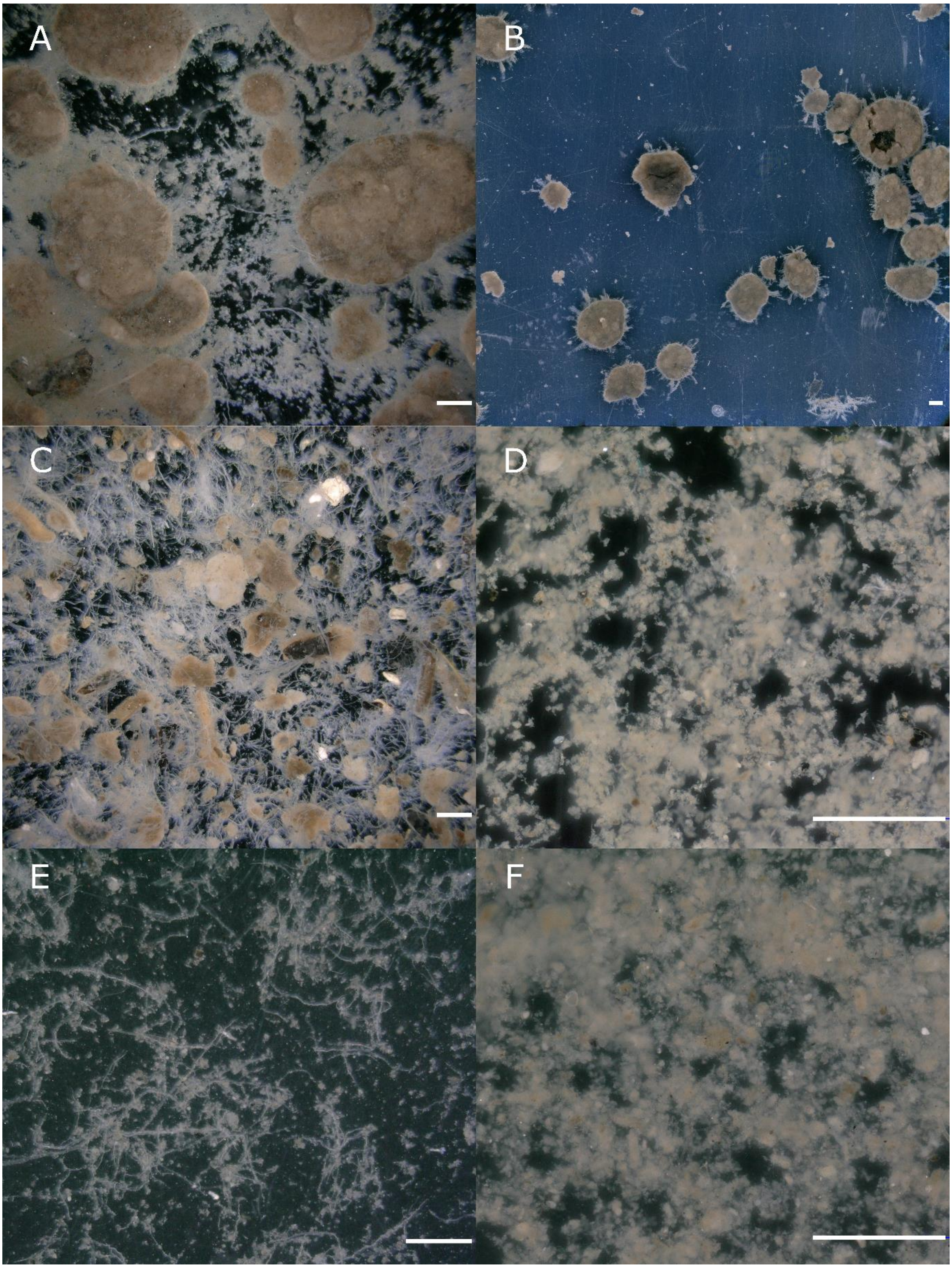

Fig. S3. Microscope images of the biomass fractions used in the assay: A) Mixed sludge, B) Large granules, C) Small granules, D) Flocculent sludge, E) Influent, and F) Crushed large granules. Size bars: 1000 µm. Note that the small granule fraction consists of a complex fiber matrix and different sizes of granules, which doesn't allow to determine the average granule diameter of this fraction.

S4. Results of the dispersion of large granules

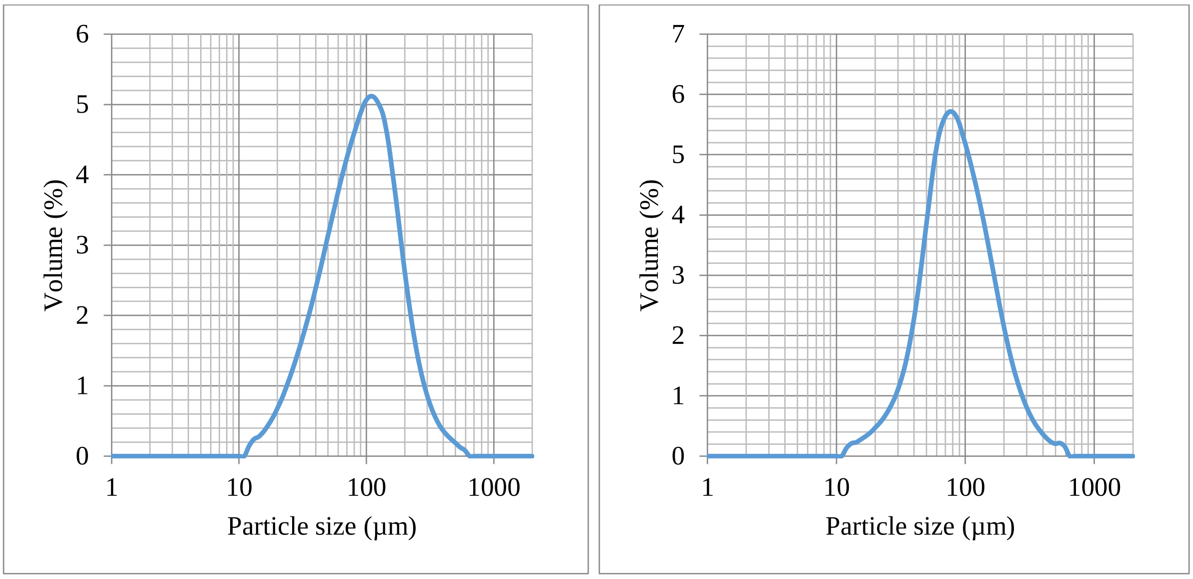

Fig. S4. Output of particle size distribution measurements of flocculent sludge and crushed large granules. A) Crushed granules; B) Flocculent sludge. The average particle size in the crushed sludge fraction was 105.1 ± 0.9 µm, and in the floc sludge fraction, 101.5 ± 0.6 µm.

S5. Daily variations of the percentage of the total reactor activity contributed by the different biomass fractions.

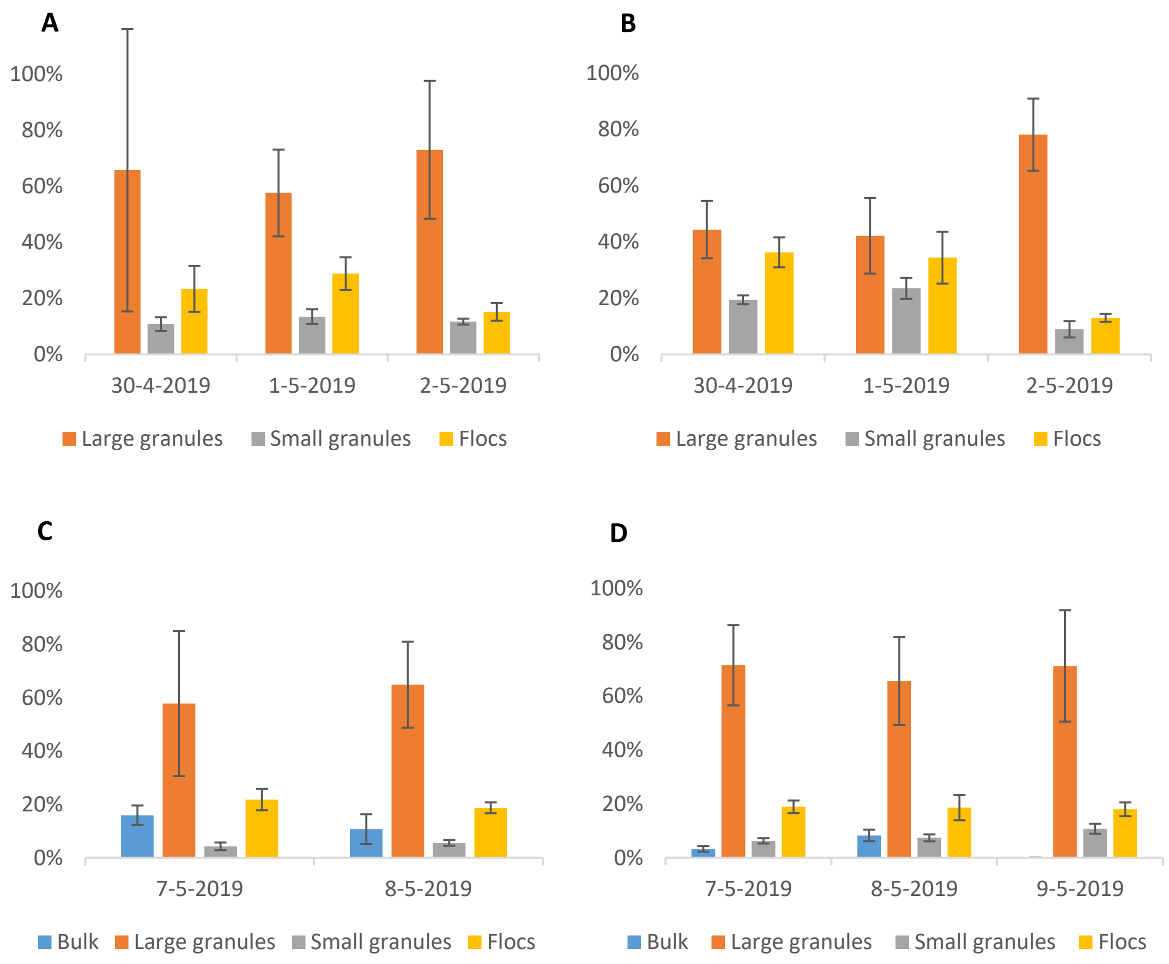

Fig. S5. Percentage contributions of enzyme activity per sludge type, in the different sampling days. a) α-Glucosidase, b) β-Glucosidase, c) Lipase, d) Protease. Note the differences in bulk activity between days for protease, and activity of granules in the case of β- glucosidase.

References

Gupta N, Rathi P, Gupta R (2002) Simplified para-nitrophenyl palmitate assay for lipases and esterases. Anal Biochem 311:98–99. doi:10.1016/S0003-2697(02)00379-2

Winkler UK, Stuckmann M (1979) Glycogen, hyaluronate, and some other polysaccharides greatly enhance the formation of exolipase by *Serratia marcescens*. J Bacteriol 138:663–670. doi:10.1128/jb.138.3.663-670.1979
